# Patient Perceptions of a Financial Resource Handout Following Gynecologic Cancer Diagnosis: A Mixed-Methods Study of Feasibility and Patient-Reported Utility

**DOI:** 10.7759/cureus.104373

**Published:** 2026-02-27

**Authors:** Arielle R Levine, Chrissy Liu, Justin Harold, Blair McNamara, Vaagn Andikyan

**Affiliations:** 1 Obstetrics and Gynecology, Yale School of Medicine, New Haven, USA; 2 Gynecologic Oncology, University of Southern Florida, Tampa, USA; 3 Gynecologic Oncology, Medical University of South Carolina, Charleston, USA; 4 Gynecologic Oncology, Yale School of Medicine, New Haven, USA

**Keywords:** feasibility study, financial stress, financial toxicity, genital neoplasms, gynec oncology, insurance coverage, multimethod study, patient education material, patient resources, perception

## Abstract

Introduction

This study aimed to evaluate and understand the perceived utility of a handout providing information about financial resources distributed to patients with diagnosed gynecologic malignancies.

Methods

We administered a survey querying patient perception of financial toxicity shortly after receiving a gynecologic cancer diagnosis and then provided a handout describing various financial resources for individuals with oncologic diagnoses. Information regarding patient demographics, oncologic characteristics, and treatment was collected. A subsequent survey was administered three to six months later, investigating the utility of the information provided in the handout. Multimethod analysis was done to evaluate the impact of the intervention.

Results

Twenty-seven participants participated in the study, with 12 participants (44.4%) having a high financial burden and 15 participants (55.6%) having a low financial burden. There were no differences in patient, oncologic, or treatment characteristics across those groups. Qualitative experience of the handout intervention was entirely positive or neutral, with no differences in intervention perception between groups. The positive responses described perceived benefits of the study for certain individuals, while the remaining neutral responses mentioned high levels of insurance coverage and experiencing support from the medical team.

Conclusion

Financial toxicity represents a meaningful source of concern and challenge for patients with gynecologic malignancies. By providing the handout, a subset of patients described meaningful anticipatory benefits, including planning for employment disruption and retirement. Patients with neutral responses to the intervention cited their comprehensive insurance coverage and existing institutional support as obviating the maximal positive impact of the handout. Further research should explore additional interventions for supporting patients with gynecologic malignancies.

## Introduction

The incidence of cancer has continued to increase over recent years, according to the American Cancer Society, with more than two million cases predicted to be diagnosed in 2024 [1]. Costs associated with cancer have also continued to increase, and the cost to the patient was estimated to total more than $21.1 billion, with $16.2 billion attributed to out-of-pocket costs and $4.9 billion as costs to patient time [1]. Rates of incidence of ovarian and cervical cancers have largely decreased over the past several decades [2,3]; however, rates of incidence of uterine and vulvar cancers have been increasing [4,5]. Demographics of gynecologic cancers are changing, with increasing diagnoses among adolescents and young adults, highlighting a shifting burden of disease to segments of the population more likely to be of reproductive age and participating in the labor force [6].

A prior study by Zeybek et al. investigated the significant financial toxicity and burden placed on patients who receive the diagnosis of a gynecologic malignancy [7]. This study highlighted the presence of financial burden to all 50 patients in the study and the various cost-coping strategies that patients were obligated to use, and some of the characteristics (lower income and higher age) associated with increased financial burden. This study raised the important question of how we can best help patients ameliorate these hardships and navigate a complex world of support services. The intricacies of medical insurance, external financial resources, and other support funds can be incredibly complex and pose a significant challenge for patients to navigate, especially when also coping with a new life-altering cancer diagnosis, particularly for patients with limited medical and financial literacy [8]. Suggestions regarding next steps to support patients facing financial burdens subsequent to all types of cancer diagnoses have called on stakeholders at all levels, including but not limited to patients, individual providers, medical systems, insurance companies, and national resources, to take on this challenge [9].

Within gynecologic oncology specifically, frameworks for addressing financial toxicity have included drawing on financial navigators, providing patients with more information, providing universal screening for the need of financial assistance, and partnering with community and outside resources to provide financial support to patients and their families [10]. However, studies examining the benefit of specific tools are still largely in progress and require more investigation to better understand how to best support various patient populations and address the burden of financial toxicity experienced by gynecologic oncology patients. Despite increasing recognition of financial toxicity, there is limited evidence evaluating the feasibility and patient perception of low cost, universally deployable educational interventions delivered at the time of diagnosis.

## Materials and methods

The study was approved by the Institutional Review Board (IRB) at Yale School of Medicine, New Haven, CT, USA (IRB approval protocol number: 2000028321). Inclusion criteria included patients 18 years and older who were presenting to the gynecologic oncology clinic at the participating institution for a gynecologic oncology diagnosis. Exclusion criteria included patients who were not presenting for a gynecologic oncology diagnosis or who were less than 18 years of age. Eligible patients presenting to the gynecologic oncology clinic at an academic center for diagnosis, treatment, and/or follow-up appointments were approached to participate in this patient education study. If interested, potential participants were consented by a member of the research team for participation in the study, and a signed informed consent was obtained. A unique research study identification number was assigned and added to a separate document for the creation of an independent key for purposes of patient study identification. A patient tracking log was also created in order to follow patients at various time points for patient survey participation.

At the initial time point of patient intake to the study, patients completed two initial surveys: The Comprehensive Score for Financial Toxicity (COST) and the Patient Survey on Attitudes and Perspectives Surrounding Cost of Care created by the Patient Advocate Foundation (PAF Survey) [11-13]. The data from these surveys were entered into a Microsoft Excel spreadsheet (Microsoft Corp., Redmond, WA, USA) without patient identifiers, which was maintained separately from the patient identification key. Patients were then provided with an educational handout (Appendix A) with information regarding various financial resources and encouragement for patients to ask their medical teams to connect them with an oncology social worker if the patient had further questions or concerns. This handout was provided to all participants regardless of patient response to the initial surveys administered.

Patient records were also accessed in order to identify patient demographic and case information (i.e., age, malignancy type, and stage at diagnosis). At a follow-up visit between three and six months subsequent to the initial intake into the study, the exact timing depended on when patients presented for follow-up in the clinic; patients were administered a subsequent questionnaire by a member of the study team (Appendix B). This questionnaire asked patients about the utility of the handout provided at the initial study intake visit. This included the opportunity for patients to provide open-ended comments about their current financial situation and/or the study material provided in this study. These results were then also entered into the data log with the corresponding patient identifier assigned at the first study visit. This was the data used for subsequent data analysis in a multi-method fashion. Valences were assigned to these comments, which were then used to contextualize the patient responses to the intervention.

Survey and handout design

This prospective interventional patient education study without controls had three surveys administered at two separate time points separated by three to six months, with a physical handout with information regarding financial resources provided at the initial time point.

The COST survey consists of 11 questions with a Likert Scale response from 0 to 4 for all questions; thus, the total points possible from this study range from 0 to 44, with 0 corresponding with no financial toxicity experienced by the study participant and 44 representing extreme financial toxicity experienced by the study participant. Patients with a score less than 22 (<22) points were classified as experiencing low financial burden, and patients scoring 22 to 44 points (≥22) were classified as experiencing high financial burden (Appendix A). The other survey administered to study participants was the PAF Survey. This survey was created by the PAF, a 501(c)(3) non-profit organization from the United States, which is involved with healthcare system navigation and assistance for patients, families, and caregivers. This survey contains 55 questions, which cover topics including patient demographics, diagnosis and health history, and financial challenges (Appendix B).

The patient education handout outlining financial resources was created as a brochure for patients that included information for patients that was created for patients receiving care from the academic center where the study was conducted. The handout provided information for patients who either did or did not have insurance coverage. It also included information regarding finding assistance for medical costs, as well as for other hardships or challenges created by the burden of an oncologic diagnosis, other expenses associated with oncology treatment, and employment impacts. The document also encouraged patients with any questions or further challenges to reach out to the medical team to request a connection with an oncology social worker.

The final survey, administered three to six months after diagnosis, was created for the purpose of this study in collaboration with PAF to assess the utility of the handout provided to patients. This included an opportunity to comment on the experience of financial hardship during the patient’s cancer treatment, any assistance or support received from the various organizations mentioned in the handout, and any other comments or information that the patient wanted to share regarding this study.

Statistical analyses

Statistical analyses were conducted using RStudio (Posit Software, Boston, MA, USA) and the Biomath functions developed by Columbia University (New York, NY, USA). Analyses were conducted to compare various factors across the financial burden score (high vs. low). Appropriate analyses were conducted based on the nature of the variable being compared across categories (continuous, categorical, ordinal), including T-tests, Wilcoxon rank-sum, Mann-Whitney U, chi-square (χ2), or Fisher's exact tests.

## Results

Twenty-eight patients consented to participate in this patient education study. One patient did not complete any of the surveys and transferred care to another care center in a different geographic location; she was excluded from all analyses. A portion of the paperwork with a subsection of the initial survey completed by one patient was unable to be located, but the rest of the initial surveys and the exit survey were completed. Four patients were lost to follow-up for completion of the final survey, and two other patients transferred care between the administration of the initial and final surveys. Twenty-seven patients completed the initial surveys and received the intervention. Twenty-one patients completed the additional follow-up survey. This indicated the feasibility of the intervention for the ability to implement the protocol, as well as the minimal additional resources required to provide this educational resource to patients eligible for the study.

The initial patient recruitment into this study was predicted to be 100 patients; however, the study was terminated early due to the reported lack of benefit being observed from the provision of the financial brochure in the preliminary data review.

The overall average age of the 27 study participants included in the analysis was 58.9 ± 12.4 years, and the average COST score was 20.3 ± 5.2.

Patient characteristics and study findings were compared across high financial toxicity burden (COST Score ≥22) and low financial toxicity burden (COST Score <22).

The comparison of high and low financial burdens as per the patients' age is listed in Table 1, with no difference in average age across high and low financial burdens (55.8 ± 11.0 years vs. 60.5 ± 13.4 years, p = 0.33).

**Table 1:** Age and Financial Burden Age-wise comparison across financial burden levels was done using Welch’s t-test. COST: Comprehensive Score for Financial Toxicity

Age & Financial Burden	Average Age ± SD (years)	Test Statistic	p-value (*<0.05)
High Financial Burden (≥22 COST Score), n = 12	55.8 ± 11.0	t = -0.99	0.33
Low Financial Burden (<22 COST Score), n = 15	60.5 ± 13.4		

The comparison of high and low financial burden as per the patients' race/ethnicity is listed in Table 2, with no difference in race/ethnicity distribution across high and low financial burden (p = 0.45). 

**Table 2:** Race/Ethnicity and Financial Burden Race/ethnicity was compared across financial burden levels using the Pearson chi-square test. COST: Comprehensive Score for Financial Toxicity

Race/Ethnicity & Financial Burden	White	Asian	Black	Hispanic	Native American	Other, Prefer Not to Say	Test Statistic	p-value (*<0.05)
High Financial Burden (≥22 COST Score), n = 12	6 (50%)	3 (25%)	2 (17%)	1 (8%)	0 (0%)	0 (0%)	χ² = 4.73	0.45
Low Financial Burden (<22 COST Score), n = 15	10 (67%)	1 (7%)	2 (13%)	0 (0%)	1 (7%)	1 (7%)		

The comparison of the high and low financial burden based on patients' insurance types is listed in Table 3, with no difference in insurance type coverage distribution across groups (p = 0.095).

**Table 3:** Insurance Type and Financial Burden Insurance type compared across financial burden levels with the Pearson chi-square test. VA: Veterans Affairs; COST: Comprehensive Score for Financial Toxicity

Insurance Type & Financial Burden	Private	Medicare + Private	Medicare	Medicaid	VA	Uninsured	Test Statistic	p-value (*<0.05)
High Financial Burden (≥22 COST Score), n = 12	9 (75%)	1 (8%)	0 (0%)	2 (17%)	0 (0%)	0 (0%)	χ² = 6.38	0.095
Low Financial Burden (<22 COST Score), n = 15	8 (53%)	6 (40%)	0 (0%)	0 (0%)	1 (7%)	0 (0%)		

The COST score for the high financial burden cohort was statistically significantly higher than the COST score for the low financial burden cohort (24.5 ± 1.8 vs. 16.9 ± 4.4, p < 0.001) (Table 4). 

**Table 4:** COST Score & Financial Burden COST score was compared across financial burden levels with the Mann–Whitney U-test. COST: Comprehensive Score for Financial Toxicity

COST Score & Financial Burden	Average COST Score ± SD	Test Statistic	p-value (*<0.05)
High Financial Burden (≥22 COST Score), n = 12	24.5 ± 1.8	U = 180	p < 0.001*
Low Financial Burden (<22 COST Score), n = 15	16.9 ± 4.4		

Comparisons across high- and low-financial burden are shown for malignancy type in Table 5, with no difference in malignancy type found distributed between the cohorts (p = 0.66).

**Table 5:** Malignancy Type and Financial Burden Malignancy type was compared across financial burden levels with the Pearson chi-square test. COST: Comprehensive Score for Financial Toxicity

Malignancy Type & Financial Burden	Ovarian	Cervical	Endometrial	Leiomyosarcoma	Fallopian Tube Carcinoma	Vulvar	Test Statistic	p-value (*<0.05)
High Financial Burden (≥22 COST Score), n = 12	2 (17%)	2 (17%)	8 (67%)	0 (0%)	0 (0%)	0 (0%)	χ² = 3.24	0.66
Low Financial Burden (<22 COST Score), n = 15	3 (20%)	1 (7%)	8 (53%)	1 (7%)	1 (7%)	1 (7%)		

The comparison of the distribution of malignancy stage across high and low financial burden cohorts is shown in Table 6, which was also similarly distributed across cohorts (p = 0.78).

**Table 6:** Malignancy Stage and Financial Burden Malignancy stage was compared across financial burden levels with the Pearson chi-square test COST: Comprehensive Score for Financial Toxicity

Malignancy Stage & Financial Burden	I	II	III	IV	Test Statistic	p-value (*<0.05)
High Financial Burden (≥22 COST Score), n = 12	7 (58%)	1 (8%)	2 (17%)	2 (17%)	χ² = 1.08	0.78
Low Financial Burden (<22 COST Score), n = 15	8 (53%)	1 (7%)	4 (33%)	2 (13%)		

The number of pre-malignancy diagnosed co-morbidities was also similar across high and low financial burden groups, with both groups having a median of three comorbidities (p = 0.71).

**Table 7:** Pre-malignancy Comorbidities and Financial Burden The number of pre-malignancy comorbidities was compared across financial burden levels with the Mann-Whitney U-test. COST: Comprehensive Score for Financial Toxicity

Pre-malignancy Comorbidities & Financial Burden	Number of Pre-malignancy Comorbidities, Median (IQR)	Test Statistic	p-value (*<0.05)
High Financial Burden (≥22 COST Score), n = 12	3 (1, 6)	U = 98.0	0.71
Low Financial Burden (<22 COST Score), n = 15	3 (1, 5)		

The high financial burden cohort was found to take a median of 2.5 non-oncology medications, while the low financial burden group took a median of two non-oncology medications, and were therefore also similar across this comparison (p = 0.88) (Table 8). 

**Table 8:** Non-oncology Medications and Financial Burden The number of non-oncology medications was compared across financial burden levels with the Mann-Whitney U-test. COST: Comprehensive Score for Financial Toxicity

Non-oncology Medications & Financial Burden	Number of Non-oncology Medications, Median (IQR)	Test Statistic	p-value (*<0.05)
High Financial Burden (≥22 COST Score), n = 12	2.5 (0.75, 6.5)	U = 86.5	0.88
Low Financial Burden (<22 COST Score), n = 15	2 (1, 7)		

Finally, the distribution of oncology treatment types each patient received, between chemotherapy, radiation, brachytherapy, or surgery, and the combinations of these treatments was also similar (p = 0.90) (Table 9).

**Table 9:** Treatment Types and Financial Burden Treatment types were compared across levels of financial burden with the Pearson chi-square test. Chemo: chemotherapy; Brachy: brachytherapy; COST: Comprehensive Score for Financial Toxicity

Treatment Types & Financial Burden	Chemo	Radiation / Brachy	Surgery	Surgery + Chemo	Surgery + Radiation / Brachy	Surgery + Chemo + Radiation / Brachy	Test Statistic	p-value (*<0.05)
High Financial Burden (≥22 COST Score), n = 12	0 (0%)	0 (0%)	2 (17%)	7 (58%)	2 (17%)	1 (8%)	χ² = 1.08	0.90
Low Financial Burden (<22 COST Score), n = 15	1 (7%)	0 (0%)	3 (20%)	7 (47%)	3 (20%)	1 (7%)		

Comparisons regarding follow-up questionnaire responses were subsequently analyzed for high and low reported financial toxicity. The comparisons of high and low financial burden are shown for the time point of follow-up in Table 10, which was not significantly different across financial burden levels.

**Table 10:** Follow-Up Time Point and Financial Burden Follow-up time points were compared across levels of financial burden with the Pearson chi-square test. N/A: not applicable or not answered; COST: Comprehensive Score for Financial Toxicity

Follow-Up Time Point & Financial Burden	3 Months	6 months	N/A	Test Statistic	p-value (*<0.05)
High Financial Burden (≥22 COST Score), n = 12	8 (67%)	3 (25%)	1 (8%)	χ² = 2.57	0.28
Low Financial Burden (<22 COST Score), n = 15	8 (53%)	2 (13%)	5 (33%)		

The comparison of the financial burden of whether they remembered the intervention is shown in Table 11. The exit survey revealed that nearly all patients (n = 20, 95.0%) remembered the brochure, and that the one patient who did not remember the brochure reported a low financial burden per the COST survey. However, this comparison was also not statistically significant.

**Table 11:** Reported Memory of Handout and Financial Burden Reported memory of the handout was compared across levels of financial burden with the Pearson chi-square test. N/A: not applicable or not answered; COST: Comprehensive Score for Financial Toxicity

Reported Memory of Handout & Financial Burden	Yes	No	N/A	Test Statistic	p-value (*<0.05)
High Financial Burden (≥22 COST Score), n = 12	11 (92%)	0 (0%)	1 (8%)	χ² = 3.58	0.17
Low Financial Burden (<22 COST Score), n = 15	9 (60%)	1 (7%)	5 (33%)		

Three patients (14.3%) found the handout to be useful and mentioned using the resources highlighted in the brochure, as shown in Table 12. All of these patients reported high financial burden; however, there was no statistically significant difference when compared across financial burden levels.

**Table 12:** Reported Use of Handout and Financial Burden Reported use of handout was compared across levels of financial burden with the Pearson chi-square test. N/A: not applicable or not answered; COST: Comprehensive Score for Financial Toxicity

Reported Use of Handout & Financial Burden	Yes	No	N/A	Test Statistic	p-value (*<0.05)
High Financial Burden (≥22 COST Score), n = 12	3 (25)%	8 (67%)	1 (8%)	χ² = 5.63	0.06
Low Financial Burden (<22 COST Score), n = 15	0 (0%)	10 (67%)	5 (33%)		

Nine patients (42.9%) out of the 21 that responded to this portion of the survey reported that they had financial worries at this three- to six-month time point after diagnosis; however, they did not proceed with any actions, as shown in Table 13. When these financial concerns were compared across the COST scores determined at the initiation of the study, the comparison bordered on significance with a p-value of 0.06.

**Table 13:** Reported Concern About Finances and Financial Burden Reported concern about finances was compared across levels of financial burden with the Pearson chi-square test. N/A: not applicable or not answered; COST: Comprehensive Score for Financial Toxicity

Reported Concern About Finances & Financial Burden	Yes	No	N/A	Test Statistic	p-value (*<0.05)
High Financial Burden (≥22 COST Score), n = 12	4 (33%)	7 (58%)	1 (8%)	χ² = 4.22	0.12
Low Financial Burden (<22 COST Score), n = 15	5 (33%)	4 (27%)	6 (40%)		

Within the qualitative data collected, a patient who denied using the handout in the survey reported that she shared the information with a friend, and that her friend found the information useful after losing her job. Another patient mentioned that the intervention “helped her plan for retirement.” Seven participants (25.0%) mentioned that they had excellent health insurance that provided excellent coverage.

Qualitative content analysis was performed on the comments provided by the patients, and valences were assigned to these open-answer survey questions. Four (19.0%) of the comments were scaled as positive, 10 (47.6%) of the comments were neutral, and seven (33.3%) of the respondents provided no additional comments. There were no negative valence comments (0.0%) expressed by the patients regarding the initiative. The comparison of comment valences is shown in Table 14, and this comparison was also not statistically significant.

**Table 14:** Comment Valence and Financial Burden Comment valence was compared across levels of financial burden with the Pearson chi-square test. N/A: not applicable or not answered; COST: Comprehensive Score for Financial Toxicity

Comment Valence & Financial Burden	Positive	Neutral	Negative	None	N/A	Test Statistic	p-value (*<0.05)
High Financial Burden (≥22 COST Score), n = 12	3 (25%)	5 (42%)	0 (0%)	3 (25%)	1 (8)%	χ² = 3.52	0.32
Low Financial Burden (<22 COST Score), n = 15	1 (7%)	5 (33%)	0 (0%)	4 (27%)	5 (33%)		

Additional quotations regarding the care and support services requested by patients are included in Table 15, which gives samples of the positive value the patients found from the intervention, reasons why they were neutral to the handout, and that there were no adverse reactions to these provisions of additional resources. This also helped provide information about patient response feasibility.

**Table 15:** Sample Qualitative Quotations by Valence

Valence	Sample Quotations	
Positive Valence	“The intervention was very helpful and helped to plan retirement.”	“(I) got laid off/lost my job and shared the brochures with a friend, which helped the friend so much.”
Neutral Valence	“(I) am well covered since 2014. Connecticut has good coverage.”	“Under Husky (State Medicaid Program), all is covered.”
Negative Valence	None	None

## Discussion

Summary of the main results

One strength of our paper is that all patients enrolled were similar in background characteristics, except for the level of financial stress they reported experiencing. Additionally, they all had either a positive or neutral reaction to the handout, thus indicating a low risk for a negative response to this handout being provided, even when it is not immediately obvious to the patients what the benefit to them of this intervention will be. The minimal burden placed on the clinic for providing this resource also shows the feasibility of this study with real potential benefit for patients, especially those with economic challenges, which are commonly found in the oncology patient population.

Our study confirmed that many patients who are undergoing gynecologic cancer treatment have financial worries regarding their treatment. We designed and tested the feasibility and patient perception of a handout detailing financial resources available to cancer patients (Appendix A) in this population. There was a notable subset (14.3%) who did find the information beneficial, while the remaining patients had a neutral to positive perception of the handout provided in the clinic. The notable lack of negative valence comments or findings indicates that this intervention has minimal risk to patients, a significant outcome for the feasibility and patient perception of the intervention. Given the negligible cost of this resource, the potential cost-benefit ratio (cost to the clinic and benefit to patients) could have highly favorable outcomes for patients at minimal cost to the clinic. This makes a very low barrier to benefit patients, even if the number needed to treat for this intervention is high. Consequently, this study establishes the feasibility of this intervention in gynecologic oncology clinical settings, as well as a positive or neutral patient perception of the handout.

This could also provide greater potential benefit to patients with more limited resources and in lower-resource settings. The proportion of patients that reported financial concerns (42.9%) was notable and spanned both patients that had initially reported high financial burden and those that had scored low on the COST survey, and was not significantly different across these groups (p = 0.12). This reflects the onset of financial burdens that can occur throughout the oncology treatment journey. Consequently, financial interventions delivered at diagnosis may function less as reactive assistance tools and more as anticipatory planning resources, especially if deployed at lower-resource settings with a more vulnerable patient population.

Feasibility assessment

The study assessed the feasibility of this intervention with respect to three of the five components of this type of study as defined by Orsmond and Cohn [14]. These three components included 1) the ability to implement the educational intervention, 2) the necessary resources needed and the ability to implement the educational intervention, and 3) the initial evaluation of participant response. For the ability to implement the educational intervention, all but one participant completed the initial part of the study and received the educational intervention. Within the context of the second component, all materials and patient education were completed by clinic staff without the requirement of additional resources after the creation of the handout. Finally, the evaluation of the patient's initial reaction demonstrated all positive or neutral responses, thus indicating the potential benefit and minimal risk of harm to patients with this educational initiative. Ultimately, these evaluations taken together provide indications that this initiative could be replicated in this or other settings. This could include potential future studies to more fully understand the target population and longer-term patient responses across the cancer treatment journey, as well as possible universal resource provision protocols to capture all possible patients that could benefit from this resource.

Results in the context of published literature

The study provided further support and confirmation of the previously published findings regarding the burden of financial toxicity and burden to patients [7,9,10,15]. Additional findings in this feasibility and patient perception study represent novel advancements in the context of published literature by examining the feasibility and patient perception of a specific resource for patients receiving treatment for a gynecologic malignancy. The development and adaptation of this tool to the clinical setting in which it was deployed demonstrates the utility of a customized financial resources handout for patients as an intervention with nominal risk and potential positive benefits. The addition of multi-method evaluation of financial burden and the experience of receiving this resource represents a further novel element of this research, thus expanding the understanding of the patient experience of this intervention. The multi-method findings elucidate likely target populations of future interventions, namely those facing unemployment, lack of insurance, and patients further along the cancer treatment journey.

Strengths and weaknesses

This study has a few important limitations that may explain the selectively beneficial impact of our handout intervention on our study population. One such limitation includes that all 28 patients (100%) had insurance, which is not representative of the general population in the United States, as only 92.1% of individuals in the United States are reported to be insured at some point in time throughout the year [16]. Universal distribution may dilute perceived benefit in well-insured populations, while targeted deployment may improve the intervention’s yield. Another limitation is that our study took place at a quaternary-care academic institution where access to many cancer-specific resources and supports, including oncology social workers and other allied professionals, is high. Our brochure intervention would likely have more impact in a setting with less ancillary support for patients. Furthermore, the level of coverage provided by the state Medicaid program was identified as covering “all” by a patient in the study (Table 4). This is particularly significant because while insurance does not alleviate all financial burden for oncology patients, it does significantly blunt these costs. Consequently, many of the patients in our study likely benefited from a markedly decreased financial burden due to their insurance coverage status, which mitigated the reported benefit of our intervention [17].

Additional limitations include that the follow-up surveys were conducted only three to six months after diagnosis, when the financial burdens of the oncology diagnosis may not have reached their peak. The exact timing of peak financial burden remains largely uncharacterized for both oncology patients generally and gynecologic oncology patients specifically. However, additional research has found a correlation between cancer diagnosis and significant financial burden bidirectionally [9]. For example, a cancer diagnosis has been correlated with the declaration of bankruptcy for a variety of cancer types, including uterine cancer [15]. Additionally, increased financial toxicity and out-of-pocket costs for medications during insurance lapses have both been associated with increased pauses in treatment [18]. Qualitative studies have also linked significant time costs with a cancer diagnosis, of which an outsized burden was associated with female oncology patients, which is particularly relevant within gynecologic oncology [19,20]. Consequently, patients in this study were as yet unaware of future financial burdens because the initial survey was administered primarily at the time of or immediately following diagnosis. This may highlight another important aspect of providing financial information as early as possible, including at the time of diagnosis, in order to help prepare patients and potentially mitigate financial burden prior to the patient experiencing adverse outcomes from both a financial and medical perspective. This could help prepare patients to consider different aspects of financial stability, including medical costs and other economic burdens: decreased ability to work, cost of transportation and parking, reliance on other household members for care, and other similar challenges. While the priming of patients regarding the potential financial toxicity of this diagnosis at the initial interview may also have influenced patient perception of this intervention, this may, in fact, represent a strength of this intervention by prompting patients to consider how they might address financial issues through other means beyond those described in the handout. Other limitations in the study design include a lack of a control group; our intervention may have provided more benefit than was captured in our study, as we were not able to assess a cohort that did not receive the handout.

Implications for practice and future research

This study did establish the feasibility of this low-barrier, potentially high-reward intervention, especially if targeted to key demographics underrepresented in our study, namely, uninsured or underinsured individuals. The responses to this survey were also broadly positive and neutral, with minimal negative perception or adverse response. Consequently, this study supports both the feasibility and minimal cost and a net benefit for patients facing the challenging situation of a new gynecologic oncology diagnosis, a notable challenge for any patient and their support system.

Future research directions include targeting this intervention to settings with more uninsured and underinsured individuals, particularly within clinical settings with more limited financial and social resources for patients. Additional investigation could compare this intervention to a control group or to other types of financial support interventions to better understand how this financial handout benefits patients. Longer-term follow-up could also further contextualize the findings, particularly by elucidating the role of these resources once the patient has experienced potentially increased financial burdens associated with the gynecologic oncology diagnosis.

## Conclusions

Financial toxicity due to gynecologic malignancies remains a significant issue for patients throughout the United States. While a universally distributed financial resource handout demonstrated limited overall utilization, a subset of patients reported meaningful anticipatory benefits. These findings suggest that low-cost educational tools may be most effective when targeted to patients at higher risk for financial toxicity or deployed within broader financial navigation strategies. This intervention deserves additional investigation to adequately support gynecologic oncology patients facing the stress and challenges associated with their diagnoses.

## Appendices

Appendix A

Financial Toxicity in Patients With Gynecologic Malignancies

**Table 16:** Financial resources handout provided to patients Credits: Vaagn Andikyan, Blair McNamara

Topics:
1. Medical Care Expenses
Insured: Paying for Care
Uninsured: Paying for Care
2. Other common expenses for which help might be available
3. Employment Impacts - Protections and Benefits
It can be a challenge to keep up with all the costs associated with diagnosing and treating cancer, even when you have insurance. Fortunately, there are organizations that can provide education, information, and assistance for patients. It can be hard to know where to start or how to make sense of all the options. This brochure provides some tips and resources organized by types of assistance: medical care, everyday expenses, and employment protections and benefits.
If you need immediate assistance or want to have a professional guide you through the process of finding help, then ask your medical team to request that an oncology social worker from Smilow contact you and/or call the Outpatient Specialty Pharmacy Services (OSPS) at 844-881-0043.
1. Medical Care Expenses
Many people need help paying for the cost of their medical care, like prescriptions, hospital bills, and health insurance premiums.
If you have insurance coverage of any type…. and you need help paying for costs directly related to your coverage, such as premiums, copays, and coinsurance, then these resources can be a helpful place to start.
Pharmaceutical Patient Assistance Programs Copay Coupon /Savings Cards (Eligibility note: these are mostly for those who DO NOT have Medicare, Medicaid, Tricare, or other federally funded plans)
Manufacturer copay assistance programs help insured patients afford prescription drugs by covering part or all of a member’s deductible and copay for certain medications.
Call Yale’s Outpatient Specialty Pharmacy Services (OSPS) at 844-881-0043, and they can help you find and apply for such programs
Search by your medication name online through the sites below, speak to your doctor, or use search engines
Needy Meds 800-503-6897
Medication Assistance Tool (mat.org)
Charitable Copay Assistance Programs
Can help with share of cost responsibilities such as premiums, deductible and cost-sharing co-pays and co-insurance of covered medication. Income eligibility requirements and availability of funding vary a lot, so look at all of them. Eligibility note: most of these are limited to Medicare, Medicaid, Tri-Care, and other federally sponsored programs, but some are open to those with any type of insurance coverage.
Cancer Care 866-55-COPAY
Good Days 877-968-7233
Healthwell Foundation 800-675-8416
National Organization for Rare Disorders 800-999-6673
Patient Access Network Foundation 866-316-7263
Patient Advocate Foundation Copay Relief Program 1-866-512-3861
Patient Services Inc. 800-366-7741
The Assistance Fund 855-845-3663
National Organization for Rare Disorders 800-999-6673
If you have Medicare and need help paying for prescription drugs, then you may qualify for the “Extra Help” program valued at about $5,100 per year. Extra Help pays for things like monthly premiums, annual deductibles, and co-payments related to Medicare prescription drug coverage. You must be receiving Medicare and have limited resources and income. You must also reside in one of the 50 states or the District of Columbia. Learn more or apply at https://www.ssa.gov/benefits/medicare/prescriptionhelp.html
If you have Medicare, you may qualify for the Medicare Savings Programs, which help pay your Part A (Hospital Insurance) and Part B (Medical Insurance) premiums and may also pay your Part A and Part B deductibles, coinsurance, and copayments. You’ll apply for Medicare Savings Programs through your state. When you apply, your state determines which program(s) you qualify for. Even if you don’t think you qualify, you should still apply.
For Medicare beneficiaries who are Disabled and seniors, it provides financial help with the costs of medicare premiums/deductibles/copays - eligibility is based on your income. Contact the State of Connecticut’s Choices Program through the Connecticut Agency on Aging, 1-800-994-9422 or www.ct.gov
Yale-New Haven Hospital and NEGM Medical Group offer free care and sliding fee scale assistance programs based on income criteria, even if you have insurance coverage - contact the billing departments for application/information.
340 B Program - provides reduced cost medications through The Apothecary at Yale-New Haven or Walgreens pharmacies, which may be able to provide reduced cost medications if you have a prescription from a Yale doctor with “340B” written on the script
If you don’t have health insurance coverage…. and you need help paying for costs directly related to your coverage, such as premiums, copays, and coinsurance, then these resources can be a helpful place to start.
Explore options for enrolling in health insurance by going through www.Accesshealthct.org if you are a Connecticut resident or marketplace.gov for non-residents. You will be screened for eligibility and Enrollment into Medicaid or other Insurance that may include financial support/discounts.
You and/or your children may be eligible for Medicaid through the Husky. Visit the website
*The Covered Connecticut may provide free health coverage if you don’t qualify for Husky Health/Medicaid. Please visit the website.
You can also contact community insurance agents - use one that is certified as a “fiduciary,” as they are mandated to work in your best interests.
Project Access -provides donated medical care for uninsured patients who live in the Greater New Haven area - you must be referred by your medical team. How/Who should they ask, or where to go to apply?
Free or discounted care may be available from Yale-New Haven Hospital or NEGM Medical Group if you are uninsured and meet income eligibility criteria. Contact the billing departments for application/information call 855-547-4584 or visit the website.
Discounted medications apps and websites can provide cash pay discounts. Compare pharmacies and sites for the best cost savings
https://GoodRx.com
https://rxsaver.com
Medication Assistance Programs are often offered by drug companies for specific products. Those without insurance may be eligible to receive drugs for free. Search by your medication name online through the sites below, speak to your doctor, or use search engines. You can also call Yale-New Haven’s Outpatient Specialty Pharmacy Services (OSPS)at 844-881-0043, and they can help you find and apply for such programs
Needy Meds https://needymeds.com 800-503-6897
Medication Assistance Tool (mat.org) 571-350-8643
2. Help with other common expenses associated with necessities related to treatment
It is not uncommon for people to struggle with other costs while undergoing treatment, such as transportation, parking, food, utilities, housing, and childcare.
Ask your medical team to refer you to your oncology social worker for applications to local or national grant programs that you may qualify for.
Use the National Financial Resource Directory to generate a list of the potential organizations that may have programs to address your needs at https://www.patientadvocate.org/explore-our-resources/national-financial-resource-directory/.
Contact Infoline “211” for referral and contact information for:
Government programs (for example: food stamps, cash assistance, rental assistance, housing, eldercare)
Community organizations (utility assistance, food programs, child care resources, transportation resources)
Faith-Based Organizations
3. Employment Impacts - Protections and Benefits
If you are currently employed or about to end your employment, there may be benefits and protections available to you during or after treatment.
Workplace Benefits that may be offered while you are employed
Family medical leave
Talk to your employer about how to apply for this resource. Explore the triagecancer.org and cancerandcareers.org sites specifically for more information on these programs and your legal rights
Financial Supplemental Benefits - Indemnity Plans
Review your benefits at work or privately purchased for any chronic disease or cancer plans that offer payment towards treatment and other costs to a qualifying diagnosis
Life Insurance
Review your benefits at work and consider rolling eligible life insurance into a private policy. With a health condition, the ability to secure other life insurance may be financially and/or medically unobtainable.
If you are employed but your treatment or condition is keeping you from work now or in the future, then you should explore disability Replacement Income and state and federal disability benefits
Speak with your employer/human resource department to review your options for available disability benefits to supplement loss of income temporarily or long-term
State Disability Insurance - check your state for these benefits.
Disability and income status may qualify you for Medicaid. Apply through the local Department of Social Services or through www.ct.gov
Social Security Administration administers retirement and disability benefits for qualified applicants. Contact the Social Security Administration to apply either online or over the phone at www.ssa.gov or 1-800-772-1213
This federally funded program will provide a monthly financial benefit. It is also a gateway to health insurance coverage through either Medicare or Medicaid. Your doctor must agree that your medical condition(s) will keep you from working for 12 months or more. Visit https://ssa.gov or call 800-772-1213 to determine eligibility and apply.
Social Security Disability Income (SSDI) is based on work credits, and the monthly benefit is based on that history. You may eventually get Medicare health insurance after a 2-year waiting period for most.
Supplemental Security Income (SSI) is not based on work credits but on financial need and has income and asset limits. You may qualify for Medicaid health insurance through the state.
If you are about to become unemployed or just lost your job, you may be eligible to extend your health insurance coverage even after your employment ends.
Compare the benefits and costs against your financial circumstances and needs
You will have 60 days to choose whether or not you elect COBRA, a temporary continuation of employment group health insurance for 18-36 months that would otherwise be lost due to certain specific events. If you need health coverage in the time between losing your job-based coverage and beginning coverage, you may wish to elect COBRA coverage from your former employer's plan. COBRA continuation coverage will ensure you have health coverage up to 18-36 months. Visit https://dol.gov or call 866-487-2365 and/or contact the employer for more information.
Your job loss may qualify you for special enrollment in a Marketplace plan. You must select a plan within 60 days before or 60 days after losing your job-based coverage. Visit https://healthcare.gov or call 800-318-8596. ( or you can enter a specific state link here). You will be screened for any financial subsidies for financial relief of costs.
If you are low-income, then you may be eligible for Medicaid ( or add state Medicaid name and info) or the Children’s Health Insurance Program (CHIP). These are free or low-cost medical coverage options offered by your state. Learn more by contacting your local Department of Social Services or state Medicaid office (www.ct.gov). Find your state at https://medicaid.gov (or enter state-specific information here with phone #)
If your spouse or parent (if you are under 26) is employed and has insurance coverage or is eligible for coverage, you may be able to gain coverage as a dependent on their plan. Loss of coverage is a qualifying event, which means you can be added to your spouse’s coverage even after enrollment has ended.
Review mortgage, car, and credit cards for any disability or job loss protection insurance to activate and provide financial relief you could be eligible for.
If you have further questions or concerns about these issues, please ask your medical team to request that an oncology social worker contact you.

Appendix B

Financial Toxicity in Patients With Gynecologic Malignancies

**Table 17:** Follow-up financial resource survey Credits: Vaagn Andikyan, Blair McNamara

Financial Resource Survey									
Circle time from the initial post-diagnosis clinic visit									
3 months	6 months	12 months							
1. Do you worry about financial problems that you have as a result of your illness (medical expenses, inability to work, etc.)?									
Y	N								
2. Do you remember receiving the attached brochure at your first visit?									
3. Which website/organization was the most helpful? Circle the number associated with the most helpful resource:									
1	2	3	4	5	6	7	8	9	10
4. Did you apply to any of the attached websites/organizations? Circle the number(s) associated with the resources you applied to:									
1	2	3	4	5	6	7	8	9	10
5. Did you receive any financial support from the attached websites/organizations? Circle the number(s) associated with the resources you received support from:									
1	2	3	4	5	6	7	8	9	10
6. Did you receive any financial support from any other organization?									
Y	N								
If yes, please list the organization and approximate amount of support:									
Please share any comments below:									
